# Brcal Defective Breast Cancer Cells Induce *in vitro* Transformation of Cancer Associated Fibroblasts (CAFs) to Metastasis Associated Fibroblasts (MAF)

**DOI:** 10.1038/s41598-018-32370-w

**Published:** 2018-09-17

**Authors:** Sreelatha K. Hemalatha, Satheesh Kumar Sengodan, Revathy Nadhan, Jithin Dev, Reshma R. Sushama, Veena Somasundaram, Ratheeshkumar Thankappan, Arathi Rajan, Neetha Rajan Latha, Geetu Rose Varghese, Arun Peter Mathew, Thara Somanathan, Priya Srinivas

**Affiliations:** 1 Cancer Research Program, Rajiv Gandhi Centre for Biotechnology, Thiruvananthapuram, Kerala India; 2 Present Address: Mouse Cancer Genetics Program, Center for Cancer Research, National Cancer Institute, Frederick, MD 21702-1201 USA; 3 Present Address: Cancer and Inflammation Program, Center for Cancer Research, National Cancer Institute, Frederick, MD 21702-1201 USA; 4 Department of Surgical Oncology, Regional Cancer Centre, Thiruvananthapuram, Kerala India; 5 Present Address: Department of Pathology, Regional Cancer Centre, Thiruvananthapuram, Kerala India

## Abstract

It is known that Cancer Associated Fibroblast (CAFs) from the primary tumor site can accompany cancer cells to a secondary site during the process of metastasis. We hypothesize that these CAFs could be transformed to an altered cell type, which can be called as Metastasis Associated Fibroblasts (MAF) in turn can support, and convoy cancer cells for metastasis. There are no published reports that have characterized and distinguished CAFs from MAF. It is well established that some of the cancer cells within the tumor mass accumulate novel mutations prior to metastasis. Hence, we speculated that mutations in the tumor suppressor gene, BRCA1, which is already reported to induce metastasis via abnormal expression of Ezrin, Radixin and Moesin (ERM), could generate MAF. In the present study, we demonstrate for the first time that CAFs isolated from primary breast cancer tissues when co-cultured with BRCA1 mutated HCC1937 cells transform CAFs to MAF *in vitro*. As expected, MAF augmented proliferation, migration and invasion along with over-expression of epithelial mesenchymal transition (EMT) markers, Ezrin and CCL5, thereby facilitating metastasis. Therefore, we inhibited Ezrin and CCL5 *in vitro* in MAF and observed that the migration and invasion abilities of these cells were attenuated. This highlights the intriguing possibilities of combination therapy using MAF inhibitors as anti-metastatic agents along with anticancer drugs, to control the metastatic spread from primary tumor site.

## Introduction

Malignant tissues sustain self-sufficiency in growth signals during cancer progression and are highly dependent on the tumor microenvironment. Fibroblasts are one of the major constituents of the tumor stroma and are crucial for tumor progression. Cancer cells originating from the normal epithelial cells can transform stromal fibroblasts in their vicinity to a myofibroblastic phenotype called the Cancer Associated Fibroblasts (CAFs)^1,2^. The tumorigenic potential of the cancer cells can be increased up to many-fold, if they are injected along with the CAFs than with fibroblasts from normal tissues i.e, NFs^3^. CAFs enhance cancer progression through their paracrine activity by the increased secretion of growth factors and cytokines, which also help in remodeling the extracellular matrix (ECM)^4–7^. Once they are ‘educated’ by cancer cells, CAFs can instigate expression of mesenchymal markers like Vimentin, αSMA^8^, FAP^9^, FSP^10^, SDF-1, MMPs^11^, HGF^12^ and TGF-β^13^.

Recent reports indicate that CAFs from the primary tumor site move through the blood stream^14^ to the distant metastatic sites along with the cancer cells and disseminate themselves. These CAFs from the primary site will undergo cell death once the fibroblast cells of the distant organ/ metastatic site take up the function of supporting tumor progression^15^. It has been reported earlier that cancer cells harboring more oncogenic mutations can have a stronger stromal interaction^16,17^. In this context, BRCA1 gene mutation that causes predisposition to hereditary breast and ovarian cancers has also been reported to increase the metastatic ability of cancer cells^18,19^. Recent reports prove that the full length BRCA1 protein (but not C terminal mutant) via its BRCT domains interacts with and inhibits the protein super family ERM, which are located at the plasma membrane, resulting in the inhibition of the motility of cancer cells^20^. Moreover, BRCA1 deficiency in cancer cells can create oxidative stress in both cancer cells and CAFs along with increased glycolysis in CAFs^21,22^. These reports have led us to hypothesize that BRCA1 deficient cancer cells can transform CAFs to an altered form, which we named as MAF that can assist in the metastasis of cancer cells. MAF may increase the tumorigenic competence of the BRCA1 defective cancer cells hence leading to rapid metastasis, which makes them a potential target in cancer therapy. In this study, we co-cultured primary CAFs (isolated from human breast cancer patient tissues) with BRCA1 deficient and proficient cancer cells *in vitro* and demonstrated that CAFs can be converted to MAFs in the presence of BRCA1 defective cancer cells. We have also shown that inhibitors to MAF specific proteins can attenuate the migration and invasion ability *in vitro*. We presume that this would also hinder the support that would be provided by the MAF to the cancer cells that disseminate from the primary site.

## Results

### CAFs induce enhanced tumorigenicity in BRCA1 deficient than proficient breast cancer cells

**Figure 1:**
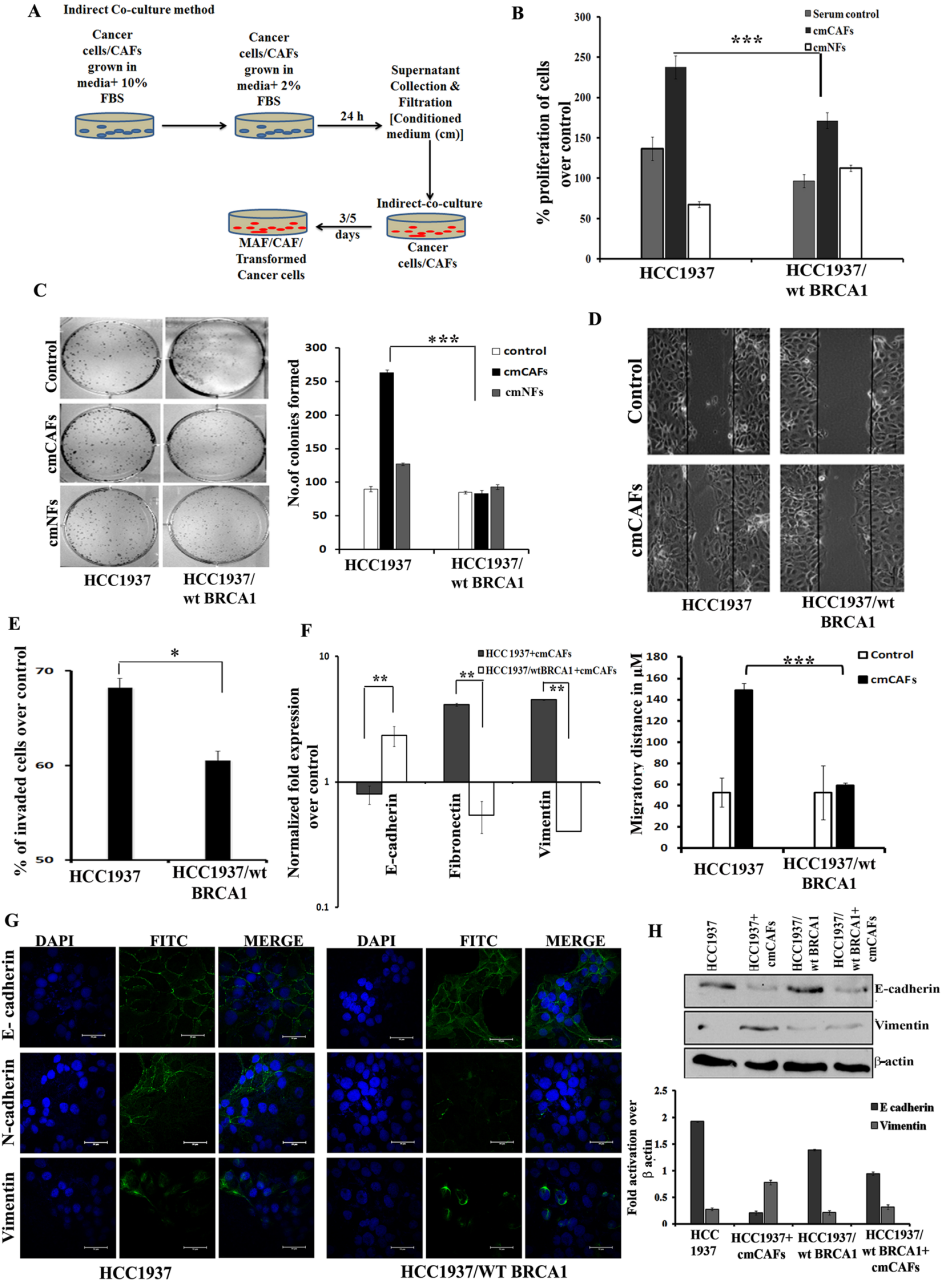
CAFs from the breast cancer tissues can increase proliferation, migration and invasion in BRCA1 deficient breast cancer cells through EMT. (**A**) Diagrammatic representation of the co-culture method used in the study. (**B**) Cell proliferation assay and (**C**) Colony formation assay (quantitation on the right panel) of HCC1937 and HCC1937/wt BRCA1 grown with cmCAFs/cmNFs. (**D**) Wound healing assay; Lower panel shows the quantitation of wound healing assay, and (**E**) Boyden chamber assay for HCC1937 and HCC1937/wt BRCA1 for the determination of invasion after co-culture with cmCAFs. (**F**) qRT-PCR analysis (**G**) Immunofluorescence analysis (scale bar 50 µm) and (**H**) Western blot analysis for the expression of EMT makers. Quantitation for the western blot analysis of figure H is indicated in the lower panel. Cells grown in RPMI 1640 with 2% FBS was used as the control in all the experiments and average of three independent experiments in triplicates are represented [*p < 0.05, **p < 0.01, ***p < 0.001, (n = 4)]. All error bars in the graphs represent standard deviation.

For determining the fibroblast-induced tumorigenicity, we co-cultured CAFs and NFs (n = 20) with the BRCA1 proficient and deficient breast cancer cells (Fig. 1A). We observed an increased expression of CAFs-specific markers, Vimentin, CD10, FAP, αSMA and FSP when compared to NFs and the epithelial marker cytokeratin 14 was found to be absent in CAFs (Supplementary Fig. S1A–D). BRCA1 defective HCC1937 exhibited a significantly higher proliferation rate than the isogenic BRCA1 proficient HCC1937/wt BRCA1 cells, when co-cultured with the conditioned medium (cm) from CAFs (cmCAFs) as evaluated by MTT and colony formation assays (Fig. 1B,C). Similarly, it was observed that the migratory and invasive capacity was more in HCC1937 than in isogenic BRCA1 proficient cells when co-cultured with cmCAFs (Fig. 1D,E).

**Figure 2:**
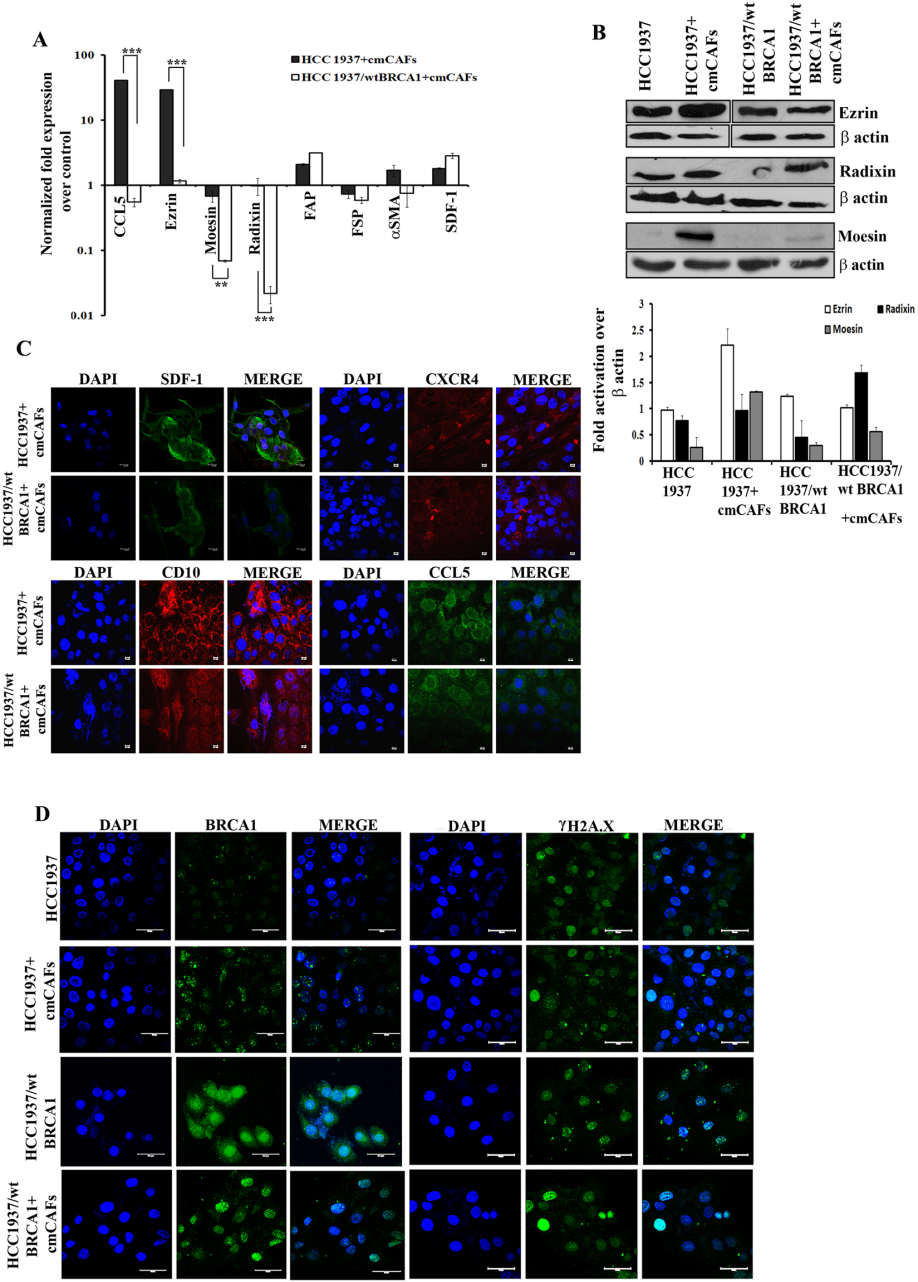
CAFs significantly induced metastatic markers in BRCA1 defective cancer cells. (**A**) qRT-PCR analysis for the expression of metastatic markers in HCC1937 and HCC1937/wt BRCA1 cells grown with cmCAFs. (**B**) Western blot analysis for the expression of metastatic markers following co-culture as indicated above. Lower panel shows the quantitation of the western blot analysis. (**C**) Immunofluorescence analysis for the localized expression of SDF-1, CXCR4, CD10 and CCL5 in HCC1937 and HCC1937/wt BRCA1 cells after indirect co-culture with cmCAFs (Scale bar 10 µm) (**D**) Expression analysis of BRCA1 and γH2A.X after indirect co-culture of breast cancer cells with cmCAF (Scale bar 10 µm). Immunofluorescence counter staining was done with DAPI. [***p < 0.001, (n = 3)]. All experiments were done in triplicates. All error bars in the graphs represent standard deviation.

These observations led us to analyze the process of EMT in these cells and we observed that cmCAFs could induce EMT more efficiently in HCC1937 than HCC1937/wtBRCA1, which was confirmed by reduction in E-cadherin and over expression of Fibronectin, Vimentin and N-, cadherin markers (Fig. 1F–H and Supplementary Fig. S6). Further, in the co-culture experiments we analyzed the changes in the expression of different genes involved in the process of cell dissemination. Approximately, 40 and 30 fold increase in the mRNA expression of the metastatic markers CCL5 and Ezrin respectively was observed in HCC1937 in comparison to HCC1937/wt BRCA1 cells, when these cells were co-cultured with the cmCAFs (Fig. 2A). The expression of ERM proteins was found to be upregulated particularly in BRCA1 defective cells when co-cultured with cmCAFs (Fig. 2B, Supplementary Fig. S6). Corroborating with the real time data, a more pronounced increase in the protein expression of angiogenic and tumorigenic markers like SDF-1, CXCR4, CD10 and CCL5, which play crucial roles in metastasis, was observed in HCC1937 cells over HCC1937/wt BRCA1 when cultured with the cmCAFs (Fig. 2C).

There was also a reduction, albeit not statistically significant, in the expression of the tumor suppressors, BRCA1 and p53 mRNA in HCC1937 than HCC1937/wt BRCA1 grown with cmCAFs (Supplementary Fig. S2A). However, it was observed that the expression of BRCA1 protein (mutant) in HCC1937 cells increased, whereas there was a reduction in the expression of wild type BRCA1 protein in HCC1937/wt BRCA1 cells (Fig. 2D). Also, the expression of the DNA damage repair protein, γH2A.X, was increased in HCC1937/wt BRCA1, but there was no change in HCC1937 cells, due to the presence of cmCAFs. Besides there was a down regulation of caveolin-1 mRNA, which is crucial for regulating cell motility. Apart from this, at the protein level, we observed a cytoplasm to cell membrane translocation of Caveolin-1 in HCC1937/wt BRCA1 and its absence in HCC1937 cells when co-cultured with cmCAFs (Supplementary Fig. S2B). These results suggest the role of CAFs in creating aggressiveness in the breast cancer cells particularly with BRCA1 mutation through EMT.

### BRCA1 mutated breast cancer cells induce the transformation of CAFs to MAF

**Figure 3:**
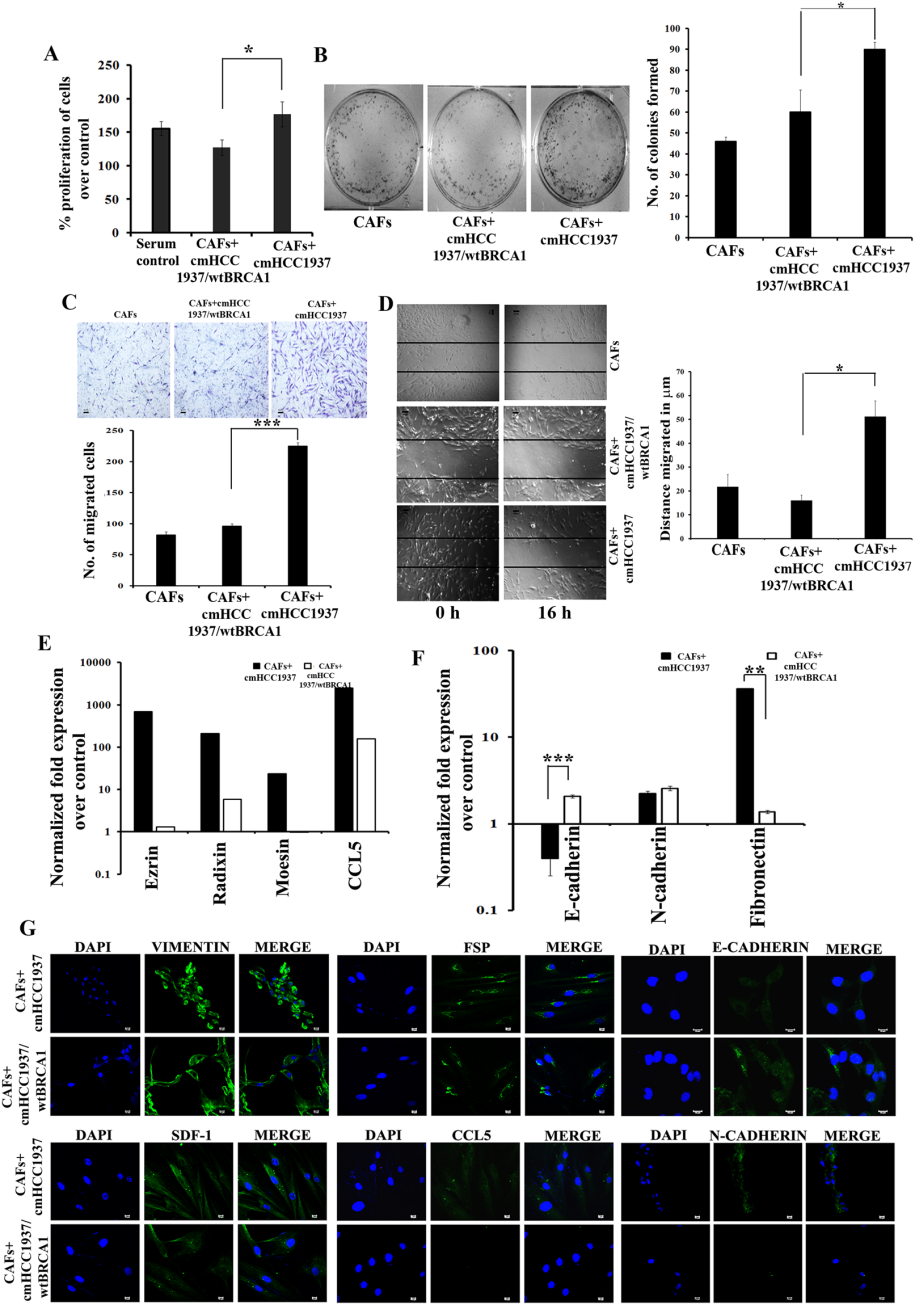
CAFs co-cultured with cmHCC1937 are characterized by their increased expression of metastatic and EMT markers. (**A**) MTT assay indicating the rate of proliferation of CAFs co-cultured with cmHCC1937 and cmHCC1937/wt BRCA1 for 3 days. (**B**) Colony formation assay of CAFs co-cultured with cmHCC1937 and cmHCC1937/wt BRCA1 for 9 days along with its quantitation in the right panel. (**C**) Transwell migration assay and its quantitation on the lower panel. (**D**) Wound healing assay of CAFs co-cultured with cmHCC1937 and cmHCC1937/wt BRCA1 and its quantitation in the right panel. (**E**) qRT-PCR analysis for the expression of Ezrin, Radixin, Moesin and CCL5 in CAFs after treating the cells with cmHCC1937 and cmHCC1937/wt BRCA1 for 5 days. Average fold expression of 4 IDC samples are represented. Individual values of each sample are represented in Supplementary Table S1. qRT-PCR expression for (**F**) E-cadherin, N-cadherin, and Fibronectin (**G**) Immunofluorescence analysis for the expression of Vimentin, FSP, E-cadherin, SDF-1, CCL5 and N-cadherin in CAFs co-treated with cmHCC1937 and cmHCC1937/wt BRCA1 (Scale bar 10 µM). DMEM with 2% FBS were used as the control for all the experiments. [*p < 0.05, **p < 0.01, ***p < 0.001, (n = 3)]. All error bars in the graphs represent standard deviation.

Having understood the role of CAFs on BRCA1 mutated breast cancer cells; we subsequently assessed the reciprocal effect of BRCA1 deficient cancer cells on CAFs. For this, we cultured primary CAFs from Infiltrating Ductal Carcinoma (IDC) (n = 4) with cmHCC1937 or cmHCC1937/wt BRCA1 for 5 days. We speculated that CAFs would be transformed to MAF only when grown with cmHCC1937 and not with cmHCC1937/wt BRCA1. Meanwhile our previous studies on the proteomic profile of BRCA1 deficient cells (HCC1937) shows that these cells express high levels of stem cell markers and other secretory proteins when compared to that of BRCA1 proficient cells (HCC1937/wt BRCA1)^23^. Since there was a significant increase in the proliferation rate of CAFs incubated with cmHCC1937 and not in CAFs incubated with cmHCC1937/wt BRCA1 (as evident from the MTT and the colony formation assay), we presumed that the CAFs might have been transformed to an altered phenotype by the secretory elements or soluble factors from the BRCA1 deficient cancer cells (Fig. 3A,B). Further, we noted a comparable increase in the migration and wound healing capability of these altered CAFs (Fig. 3C,D). These results were confirmed using cm from BRCA1 wild type MCF-7, MDA-MB 231 cells and BRCA1 defective MX1 cells (Supplementary Fig. S2C). We were unable to observe any notable morphological difference between CAFs treated with cmHCC1937 and cmHCC1937/wt BRCA1 (Supplementary Fig. S2D). Also, there was no significant changes in the percentage of CD10 population in CAFs grown with cmHCC1937/ cmHCC1937/wt BRCA1 (Supplementary Fig. S3A). These results indicate that gene mutations that affect the cell motility in cancer cells can transform CAFs to MAF.

### MAF has an augmented expression of metastatic and EMT markers

Duda *et al*.^14^ tracked the CAF cells after tagging them with GFP and showed that CAF (that is equivalent to MAF in the present *in vitro* study) are moving along with cancer cells to the metastatic sites. These MAF cells have higher migration rates and higher expression of metastatic proteins like Ezrin and CCL5. Concurrently, in our study, we found that there was a profound increase in the mRNA expression of CCL5, Ezrin, Radixin and Moesin in CAFs co-cultured with the cmHCC1937, particularly from IDC tissue samples (Fig. 3E and Supplementary Table S1). Besides, there was an augmented expression of EMT markers with reduction in E-cadherin and induction of Fibronectin, in CAFs co-cultured with the HCC1937 when compared with those co-cultured with HCC1937/wt BRCA1 (Fig. 3F). The mRNA levels of Caveolin-1, BRCA1 and p53 were down regulated in CAFs grown with HCC1937/wt BRCA1 (Supplementary Fig. S3B). The CAFs might have undergone EMT to generate MAF as there was an induction in mesenchymal proteins, CCL5 and N-Cadherin with concomitant decrease in E-Cadherin (Fig. 3G). Thus, BRCA1 mutation in cancer cells can impart increased mesenchymal phenotype in CAFs.

### MAF possess enhanced tissue remodeling ability

**Figure 4:**
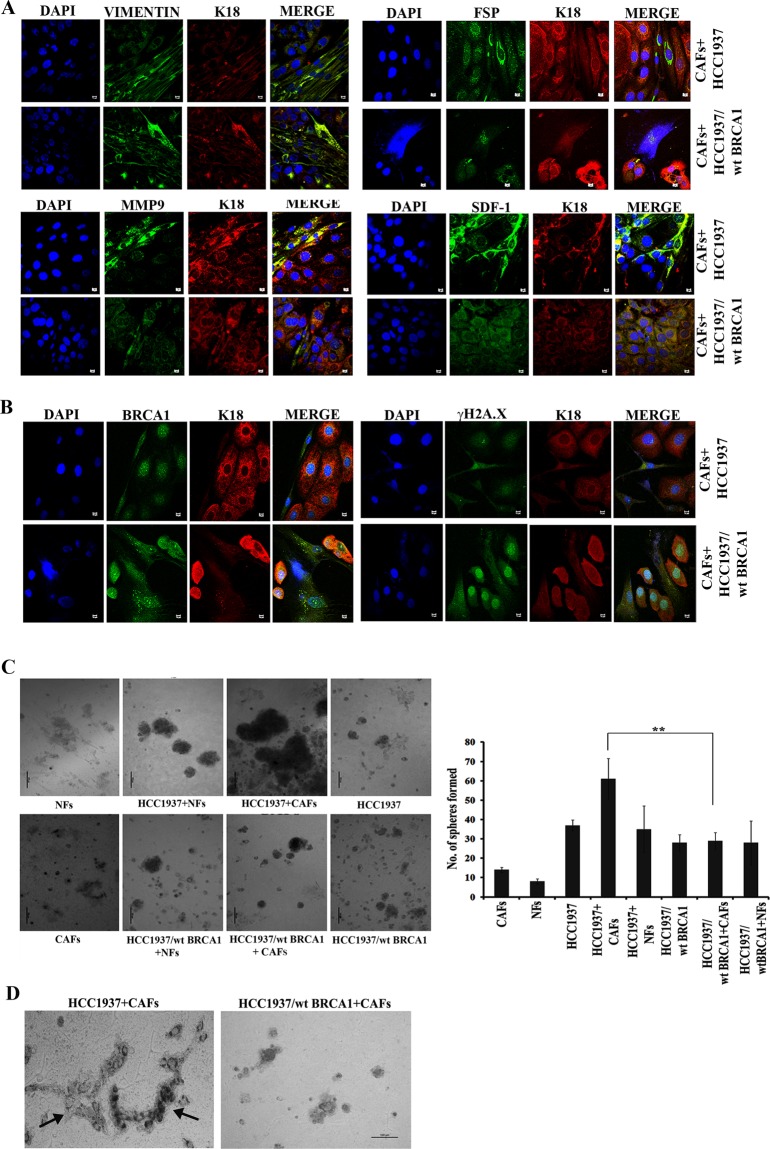
Metastasis associated protein expression in CAFs directly co-cultured with HCC1937 and HCC1937/wt BRCA1. Immunofluorescence analysis for the expression of (**A**) Vimentin, FSP, MMP9 and SDF-1 after direct co-culture of CAFs and breast cancer cells and (**B**) BRCA1 and γH2A.X expression after direct co-culture of breast cancer cells with CAFs. Cytokeratin 18 (K18) is used to distinguish CAFs cells from cancer cells (Scale bar 10 µM). (**C**) Phase contrast images of the spheres formed after 3D culture of NFs, HCC1937 with NFs, HCC1937 with CAFs, HCC1937, CAFs, HCC1937/wt BRCA1 with NFs, HCC1937/wt BRCA1 with CAFs, and HCC1937/wt BRCA1 and the right panel shows the graphical representation of number of spheres formed in the above experiment (**D**) Phase contrast images of duct like structures formed after 3D co-culture of breast cancer cells with CAFs. Black arrows indicate the ducts formed in co-cultures. Images were taken after 20 days of co-culture in a scaffold with intermediate change of medium after every three days. [**p < 0.01, (n = 3)]. All error bars in the graphs represent standard deviation.

We further performed a direct co-culture of CAFs and cancer cells *in vitro* to substantiate the above findings with indirect co-culture. The expression of different metastatic associated protein markers like FSP, MMP9 and SDF-1 are increased in CAFs directly co-cultured with HCC1937 than those co-cultured with HCC1937/wt BRCA1 (Fig. 4A). Similar to the observations in cancer cells co-cultures with cmCAFs; we observed that in direct co-culture also the foci formation by, γH2A.X was higher in HCC1937/wt BRCA1 than in HCC1937 cells (Fig. 4B). Further, we confirmed the increased expression of Ezrin, Radixin, Moesin and CCL5 in Lymphovascular Infiltrating (LIF) tumor tissues when compared to the Ductal Carcinoma *In Situ* (DCIS) and the normal breast tissues by immunohistochemical analysis of Formalin fixed paraffin embedded (FFPE) tissue sections (Supplementary Fig. S3C). Additionally, a 20 day direct 3D co-culture in a scaffold mimicking the ECM, revealed that both the number and size of the spheres formed by HCC1937 with CAFs were significantly higher than HCC1937/wt BRCA1 cells with CAFs/NFs or HCC1937 with NFs (Fig. 4C). It is interesting to note that HCC1937 with CAFs could form duct like structures when compared to HCC1937/wt BRCA1 with CAFs (Fig. 4D). This suggests that in the presence of CAFs, BRCA1 mutated cancer cells in aggressive cancers have the potential to induce tissue remodeling during tumor progression.

**Figure 5:**
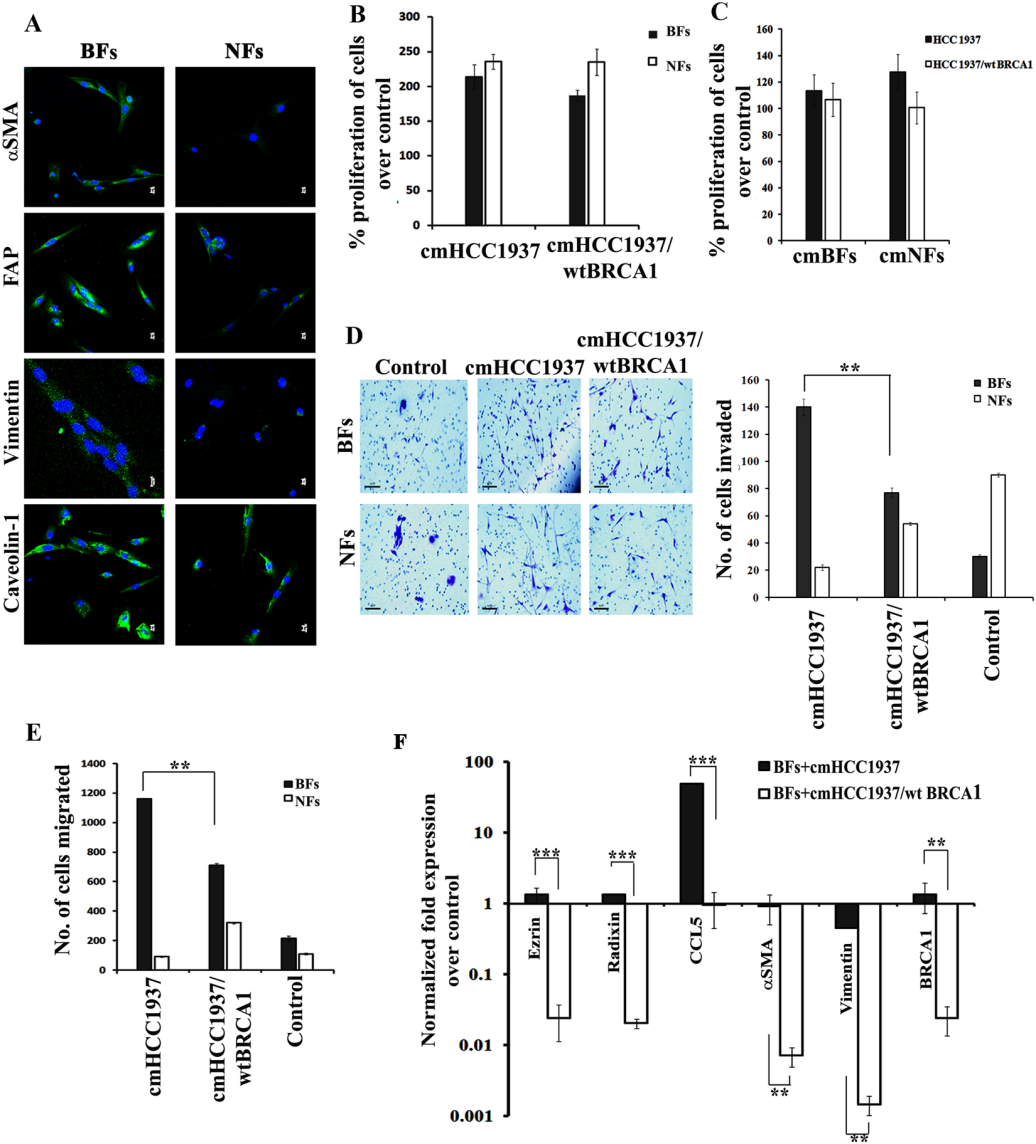
Changes observed in BFs and NFs from the adjacent tissue with BRCA1 mutated breast cancer cells. (**A**) Immunofluorescence analysis for the expression of αSMA, FAP, Vimentin and Caveolin-1 in BFs and NFs (Magnification: 60X). Proliferation analysis of (**B**) BFs/NFs in presence of cmHCC1937 and cmHCC1937/wt BRCA1 (**C**) HCC1937 and HCC1937/wt BRCA1 in presence of cmBFs/cmNFs by MTTassay. (**D**) Invasion (right panel represents the quantification of the invasion assay) and (**E**) Migration assay of BFs in the presence of cmHCC1937 and cmHCC1937/wt BRCA1 by transwell migration method. (**F**) qRT-PCR analysis for the expression of metastatic markers in BFs after co-culturing BFs with cmHCC1937 and cmHCC1937/wt BRCA1. BFs/NFs grown in DMEM with 2% FBS were used as the control. All the experiments were done in triplicates and standard error was calculated by taking the SD values of the average. [**p < 0.01, ***p < 0.001]. All error bars in the graphs represent standard deviation.

We have also noticed that the fibroblast cells from a benign breast tissue (BFs), which was positive for αSMA, FAP, Vimentin and Caveolin-1, did not show significant difference in the growth rate compared to NFs when grown with cmHCC1937 or cmHCC1937/wt BRCA1 (Fig. 5A,B). Also, there was no significant increase in the proliferation rate of HCC1937 cells grown in cmBFs when compared to cmNFs (Fig. 5C). Interestingly, an extensive increase in the number of invading and migrating BFs was observed in presence of cmHCC1937 when compared to BFs co-cultured with cmHCC1937/wt BRCA1 (Fig. 5D,E). But in contrast, we did not observe much difference in the expression of Ezrin, Radixin, αSMA, Vimentin and BRCA1, except for CCL5, which has an extensively increased fold activation in BFs after co-culture with cmHCC1937 (Fig. 5F). This suggests that BFs may have the potential to induce metastasis by transforming themselves if the environment is favorable and also that the CAFs and BRCA1-mutated cancer cells have reciprocal regulatory effects on one another.

All these observations compelled us to consider the existence of a group of CAFs, with an altered phenotype (embedded among the primary CAFs), that is conditioned by BRCA1 mutated cancer cells. These ‘altered’ CAFs could then facilitate tumor metastasis. We named the altered CAFs co-cultured with HCC1937 and HCC1937/wt BRCA1 as MAF and CAF respectively.

### Inhibitors targeting CCL5 and Ezrin impede the alteration of CAFs to MAF

**Figure 6:**
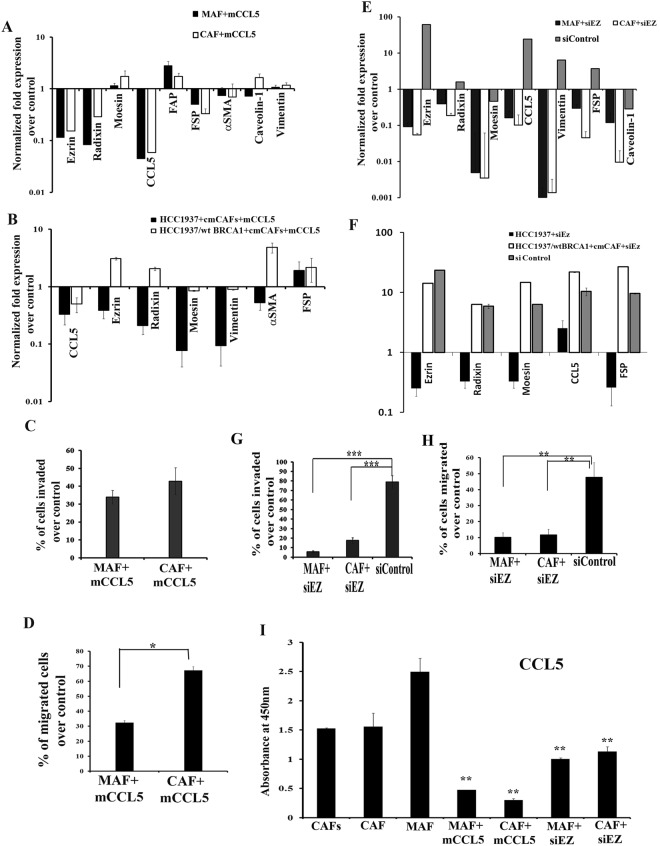
Inhibitors targeting CCL5 and Ezrin attenuated the metastatic potential of MAF. qRT-PCR analysis for the mRNA expression of metastasis associated genes after treating (**A**) MAF and CAF with metCCL5 and (**B**) HCC1937 and HCC1937/wt BRCA1 in cmCAFs with metCCL5. (**C**) Invasion assay and (**D**) Migration assay of MAF and CAF treated with metCCL5 using transwell inserts. qRT-PCR analysis of the mRNA expression of metastasis associated genes in (**E**) MAF and CAF and (**F**) HCC1937 and HCC1937/wt BRCA1 in cmCAFs transiently transfected with Ezrin siRNA plasmid. (**G**) Invasion assay and (H) Migration assay of MAF/CAF after transiently transfecting with Ezrin siRNA plasmid. MAF/CAF and HCC1937/HCC1937/wt BRCA1 in cmCAFs without inhibitors were considered as control for all the experiments. (**I**) Secretory levels of CCL5 in CAFs, CAF, MAF and MAF/CAF after CCL5/Ezrin inhibition by ELISA analysis. All error bars in the graphs represent standard deviation.

We used specific inhibitors of various proteins that are differentially regulated between CAFs and MAF to inhibit CAFs-MAF transformation and subsequently impede metastasis. CCL5 and Ezrin were found to be extensively over expressed in MAF; thus, we intended to inhibit them to unveil the role of these proteins in metastasis. Analysis of transcriptome data sets from TCGA (The cancer genome atlas) using Oncomine and cBioPortal, confirmed the inverse correlation between BRCA1 and CCL5/ Moesin in breast cancer (Supplementary Fig. S4A). A recombinant protein inhibitor metCCL5, could inhibit CCL5 in both HCC1937/wt BRCA1 and HCC1937 (Supplementary Fig. S4B). We observed that the CCL5 inhibition could create a slight, however, insignificant difference in the proliferation rate of cancer cells (Supplementary Fig. S4C). metCCL5 could significantly down regulate the mRNA expression of different genes like Ezrin, Radixin, FSP, and αSMA, in addition to CCL5 in CAFs co-cultured with both cmHCC1937 and cmHCC1937/wt BRCA1 (Fig. 6A). However, a substantial decline in the mRNA expression of Ezrin, Radixin, Moesin, Vimentin and αSMA was observed in HCC1937 cells compared to HCC1937/wt BRCA1 when treated with metCCL5 in the presence of cmCAFs (Fig. 6B). The *in vitro* invasive and migratory potential of both MAF and HCC1937 cells grown with cmCAFs reduced when CCL5 was neutralized with metCCL5 (Fig. 6C,D and Supplementary Fig. S4D).

Additionally, Ezrin was transiently inhibited in cancer cells by transfecting with siRNA plasmid against Ezrin. This was confirmed by qRT-PCR analysis (Supplementary Fig. S4E). A significant reduction in the mRNA expression of genes, Ezrin, Radixin, Moesin, CCL5, Vimentin, FSP and Caveolin-1 were observed in both the CAFs and MAF (Fig. 6E). Also, Ezrin inhibition could reduce the expression of the above mentioned genes only in HCC1937 cells and not in HCC1937/wt BRCA1 (Fig. 6F). Consistent with the previous observation with metCCL5, Ezrin inhibition also caused a considerable reduction in the migratory and invasive potential of CAFs co-cultured with cmHCC1937 *in vitro* (Fig. 6G,H). The level of secreted CCL5 was also found to be reduced when both CCL5 and Ezrin were inhibited separately in both CAF and MAF (Fig. 6I). Thus, our results open up avenues to the possibility of targeting CCL5 and Ezrin as a strategy to inhibit the effect of MAF on metastasis, predominantly in BRCA1 defective breast cancers.

## Discussion

One of the major cellular components of tumor stroma/ microenvironment is the fibroblast, which is crucial for tumor progression. As reviewed by Kalluri *et al*.^24^, the fibroblast population behaves in a context-dependent manner during different stages of tumor progression. Several gene modifications including BRCA1 mutations in the cancer cells facilitate cancer progression and metastasis^25,26^. Based on this information, we expected that mutations of BRCA1 might subsequently result in the adaptation of cancer cells by reciprocally altering their stroma to facilitate dissemination to distant sites. After the report by Lisanti *et al*.^21^ on BRCA1 mutation in cancer cells with corresponding Caveolin-1 inhibition, along with induction of glycolysis in CAFs, there are very few reports published on the changes attributed to CAFs by a single gene mutation in cancer cells^21^. Wild type BRCA1 interacts with ERM proteins which are crucial in inhibiting the motility of cancer cells, whereas mutated BRCA1 protein enhances cell motility and migration *in vitro*^20^. In this study, we depict the crosstalk between CAFs and BRCA1 deficient breast cancer cells *in vitro*, which results in the transformation of CAFs to an altered phenotype of CAFs, which we named as the MAF.

The proliferative potential induced by CAFs on cancer cells is already well known and explained in many cancers^27,28^. In our study, genetically unstable BRCA1 deficient breast cancer cells exhibited enhanced proliferation, migration and invasion compared to BRCA1 wild type breast cancer cells, when co-cultured with cmCAFs. The increased EMT in epithelial cancer cells by TGF-β secretion from CAFs has been well demonstrated^29–31^, which could also contribute to stemness^32^ in cancer cells. Here, we have seen that in presence of CAFs, there is a strong loss of epithelial markers and a modest increase in mesenchymal markers in BRCA1 mutant but not wild type cancer cells confirming the onset of EMT. This can further be correlated with the direct inhibition of EMT markers in the presence of wild type BRCA1, by promoting the proteins TWIST and Slug^18,32^. Moreover, a recent study divulges the role of exosomal exchange of miRNAs between CAFs and cancer cells resulting in tumorigenic induction via EMT and stemness^33,34^.

It has been reported that the BRCT domain of BRCA1 colocalizes with Ezrin, Moesin, Radixin and F-actin in the plasma membrane and control cell motility^20^. The cytoskeleton linker Ezrin can activate the chemokine (c-c motif) ligand 5 (CCL5) induced invasive ability in cancers^35,36^. In connection with this, in our study, co-culture of cmCAFs with HCC1937 cells but not HCC1937/wt BRCA1 cells, exhibited elevated expressions of Ezrin along with the overexpression of CCL5, which may be the reason for enhanced invasion and migration. The increased protein expression of Moesin and Radixin could be due to increased stability of the respective mRNAs. Further, direct and indirect co-culture studies with CAFs, showed an increased γH2A.X foci formation and BRCA1 protein expression in HCC1937/wt BRCA1 than HCC1937, indicating an efficient DNA damage response that might be occuring in BRCA1 wild type cells. Though there was reduction of mutant BRCA1 mRNA, its protein was expressed in cancer cells when HCC1937 were co-cultured with cmCAFs probably due to increased stability of mutant BRCA1^37^.

From the first part of the study we could substantiate the role of CAFs in inducing the metastatic ability exploiting the BRCA1 deficiency in breast cancer cells. Outschoorn *et al*., 2014, reported that BRCA1 mutated cancer cells induce oxidative stress and enhance glycolysis in CAFs^38^. Besides this, the translocation of CAFs from the primary tumor site to the distant metastatic site, was reported in metastatic colorectal, brain and liver cancers^14,15,39–41^. In this context, we hypothesized that the transformation of CAFs to an altered phenotype could induce metastatic changes in cancer cells and accompany them during metastasis. Here, we speculate that co-culturing CAFs with HCC1937 but not HCC1937/wt BRCA1, could transform CAFs to MAF owing to the strong interaction between mutant BRCA1 protein and metastatic ERM proteins and CCL5.

As hypothesized, an enhanced proliferation, migration and invasion was observed when CAFs were treated with cmHCC1937 indicating that CAFs might have transformed to MAF. Moreover, we have observed an extensive induction of CCL5, Ezrin, Moesin and Radixin in MAF when compared to CAF. These changes were attributed mostly to MAF generated from IDC samples. This also indicates that the origin of CAFs plays a crucial role along with mutation status of cancer cells. The increase in the expression of EMT marker proteins in MAF may aid these cells in accompanying the cancer cells to distant metastatic sites. Concurrent with Ezrin and CCL5 expression studies, we observed an increased number of spheres and duct formation when CAFs are grown with HCC1937 as compared with HCC1937/wt BRCA1 in 3D co-culture with CAFs. This indicates the plasticity and aggressiveness attained by the tumors by CAFs. The increase in the number of spheres, reciprocate tissue remodeling ability of BRCA1 mutated cells in the presence of CAFs.

We could not observe a noticeable increase in the proliferative induction on BRCA1 defective cancer cells by BFs or vice versa when co-cultured together. However, these fibroblasts from benign hyperplasia could respond to cm from BRCA1 defective breast cancer cells with high induction of CCL5. These observations indicate that the increased migration and invasion seen in BFs by the presence of cmHCC1937 could be due to up regulation of CCL5.

The increased expression of metastatic marker proteins CCL5 and Ezrin in MAF is of immense significance. The role of CCL5 secreted by CAF in cancer metastasis is already well established^42^. It is capable of inducing the ERM proteins at the plasma membrane and activates Rho to stimulate EMT in breast cancers^43^. We have already observed an induction of Ezrin and CCL5 proteins in HCC1937 cells co-treated with cmCAFs and also in MAF. ERM protein complex when activated, inhibits cell adhesion and enhances migration and invasion^44^. Ezrin is found to be more crucial than Moesin and Radixin for tumor induction^45^ and if Ezrin is inhibited, Radixin and Moesin will also be inhibited^46^. It has been reported that when Ezrin was silenced, CCL5 induced cell motility and invasiveness was also inhibited indicating the potential role for Ezrin in the process of CCL5 induced breast cancer cell metastasis^47^.

Therefore, in our study we inhibited the two crucial markers of MAF, Ezrin and CCL5, separately to see whether the metastatic potential of MAF can be attenuated. Both these inhibitors could reduce the expression of CCL5, Ezrin, Radixin and FSP in both CAF and MAF, the reduction in CCL5, Ezrin, Radxin, Moesin, Vimentin and α-SMA is distinctly high in BRCA1 mutated and wild type cells. As indicated by the expression studies, the wild type BRCA1 is neutralizing the effect of inhibitors when co-cultured with the CAFs. An effective reduction in the migratory and invasive ability along with reduction in the secretory levels of CCL5 protein in MAF than CAF was also observed with both the inhibitors, suggesting the possibility to attenuate the crosstalk between MAF and BRCA1 mutated cancer cells.

To conclude, in this study, we provide proof of principle for the concept that novel mutations in key genes that control cancer cell motility and their cumulative effect on CAF can induce metastasis. In the present study, we have focused only on the BRCA1 mutations in the breast cancer cells and their effect on CAF. Even in cases where the BRCA1 phenotype remains normal, mutations in other key genes that control motility might affect the stroma, resulting in cancer progression. When p53 is mutated, its inhibitory on the adhesion molecule EpCam is lost this in turn will initiate migration of cancer cells from the primary site^48^. Similarly BRAF mutation could induce motility in skin cancers^49^ (Supplementary Fig. S5).

Hence, we propose that as cancer cells acquire defects in the metastasis-associated genes, inhibiting MAF would be effective in preventing the transformation of stromal fibroblasts. We identify for the first time that Ezrin or CCL5 inhibitors can target CAFs and prevent their transformation to MAF, especially in BRCA1 mutated conditions. This concept, once validated by *in vivo* pre-clinical studies, would pave the way for future clinical trials involving combination therapy using inhibitors of MAF specific proteins like CCL5 or Ezrin, along with classical anticancer drugs.

## Materials and Methods

### Cell culture

HCC1937, breast adenocarcinoma, triple negative breast cancer cell line with 5382insC mutation in BRCA1 that leads to the synthesis of a C-terminal truncated BRCA1 protein and its isogenic cell line HCC1937/wt BRCA1 where BRCA1 cDNA was subcloned in HCC1937 using pBabe-hygro plasmid were used in the study. MX1 is a triple negative breast carcinoma cell line, consisting of 3363delGAAA in BRCA1 gene that would result in a frame shift mutation, which is predicted to introduce a chain termination and truncate the protein at residue 999. The cell is also burdened with two single nucleotide polymorphisms in BRCA2 (BRCA2 16864 A > C and BRCA2 221847 A > G). These cells were grown in RPMI (PAN Biotech, Dorset, UK) medium supplemented with 10% FBS (PAN Biotech, Dorset, UK). We have also analyzed Human breast epithelial adenocarcinoma cell lines, MCF-7 and MDAMB-231 having wild type BRCA1. These cells were grown in DMEM (GIBCO, California, USA) medium supplemented with 10% FBS. The authenticity of the cell lines has been verified by STR profiling.

### Isolation of fibroblasts from human breast tissues

CAFs and NFs were isolated from IDC and DCIS tumor tissues and the adjacent non-malignant part of the same resected tissue samples respectively after obtaining the informed consent from the patients and ethical clearance from Institute Human Ethics Committee, Regional Cancer Centre (RCC) (HEC No. 11/2012), and RGCB (IHEC/01/2012/05), Thiruvananthapuram, Kerala, India and all the experiments were performed following the relevant guidelines and regulations of the institute. Briefly, tissues were washed with 1X PBS, digested with collagenase (Stem Cell Technologies, MA, USA), separated using differential centrifugation method and CAFs were grown in DMEM supplemented with 10% FBS and 2X antibiotic/antimycotic (Invitrogen, California, USA).

### Immunofluorescence

Cells grown on coverslips in 24 well plates were fixed with 4% paraformaldehyde (Sigma, USA), blocked with 3% BSA (Sigma, USA) and incubated with primary antibodies (Supplementary Table S2) overnight. Fluorescence conjugated secondary antibodies specific for primary antibodies were added and incubated at room temperature for 1 h. Cells were then counterstained with DAPI (Invitrogen, California, USA) for nucleic acid staining and mounted to observe under a confocal microscope (Nikon).

### Flow cytometry

Cells (1 × 10^5^) were grown in the 6 well dishes, trypsinized and treated with specific antibodies (fluorescence conjugated) for 45 m in ice and centrifuged. Cell pellets were then strained using 40 µm cell strainer (BD Falcon, MA, USA) and analyzed by flow cytometry (BD FACS Aria, CA, USA). The data was then analyzed using FACS Diva software.

### Western blot

For western blot analysis, the whole cell lysates isolated from the cells using RIPA buffer was quantified using Bradford assay and separated using SDS PAGE using appropriate percentage of acrylamide gel and then transblotted to a nitrocellulose membrane. The blots incubated with primary and suitable secondary antibodies were visualized with a chemiluminesence reagent (Thermo scientific). The antibodies used have been detailed in the Supplementary table S2.

### RNA isolation and Quantitative Real Time PCR (qRT-PCR)

Total RNA was isolated using the trizol reagent. The ethanol cleared RNA was quantified using nanospectrophotometer (Nanodrop, DE, USA) and 1 µg of total RNA was converted to cDNA using High capacity cDNA reverse transcription kit (ABI) as per manufacturers protocol. Relative and absolute quantification of the expression of different genes were done by Sybr green real time PCR kit (Kappa biosystems, MA, USA) using real time PCR analyzer (ABI, CA, USA). The primers used have been listed in the Supplementary table S3. The expression levels of the genes were determined through qRT-PCR, normalised to glyceraldehyde-3-phosphate dehydrogenase (GAPDH). The log2 (ratio of mRNA level) value is the logarithm of normalised mRNA expression level (2^−∆∆Ct^) of the samples relative to the normalised mRNA expression level (2^−∆∆Ct^) of GAPDH.

### Co-culture studies

In the indirect co-culture method, cancer cells were either grown with cm from CAFs from 3 days or CAFs were grown with cm from HCC1937 or cmHCC1937/wt BRCA1 for 5 days. For collecting cm, cells (cancer cells or CAFs) grown in their respective medium were washed with 1X PBS after they have attained 70% confluency and then incubated with 2% culture media with FBS. After 24 h, the supernatant was collected, centrifuged to remove debris, filtered using 2 µm pore size syringe filter (Millipore, Boston, UK) and freeze stored until use. In direct co-culture CAFs/NFs and cancer cells (HCC1937 and HCC1937/wt BRCA1) were grown together in dishes and used for various experiments.

### Cell proliferation assay

The cancer cells (5000 cells/well in a 96 well plate) incubated with the cm from CAFs or vice versa were used for cell proliferation assay. Cancer cells and CAFs were allowed to grow in this medium for 3 or 5 days respectively and then analyzed using the MTT reagent (USB, OH, USA) colorimetrically at 570 nm. The respective cells grown in 2% culture media with FBS served as control.

### Colony formation, wound healing, migration and invasion assays

For colony formation assay 1000 cells per well in a six well plate, were seeded and cultured with cm as mentioned above and incubated for 9 days with the addition of fresh cm every third day. The colonies were then fixed with 2% formaldehyde, permeabilized using 100% methanol and stained with 0.5% crystal violet stain solution and the number of colonies was counted. The colonies containing more than 50 cells were counted and quantitated.

In wound healing assay a wound was created with the aid of a 200 µl pipette tip in the confluent cells in plate. The cells were then incubated with the respective cm as indicated above and the wounds were allowed to heal for 16 hrs. The healing time was noted and distance, the cells migrated was measured with ProgResCapturePro 2.8.8. software.

For transwell migration assay, the cells pretreated with cm as indicated above were seeded on to 8 µm pore size inserts (BD Corning) with 2% culture media with FBS. The lower chamber contained respective medium with 10%FBS and incubated for 24 h for cell migration. The cells in the inserts were then fixed with 2% formaldehyde, permeabilized with 100% methanol and stained with 0.5% crystal violet solution followed by examining under a compound microscope. The same method was followed for invasion assay except that the inserts used were coated with GFRM (Growth Factor Reduced Matrigel (BD Corning, MA, USA)).

### 3D morphogenesis analysis

The assay was performed using algimatrix scaffold based system from ABI technologies, CA, USA as per the protocol. To the activated scaffolds in the plate, CAFs and HCC1937 or HCC1937/wt BRCA1 were added in 1:2 ratio in 2% culture media with FBS and incubated for 20 days with regular change of medium on every three days. The spheres, thus formed, were analyzed for their difference in size and number using a phase contrast microscope (Olympus, Tokyo, Japan). Further, the spheres were isolated after dissolving the scaffolds as per the protocol.

### Immunohistochemical analysis

The paraffin embedded tissue (FFPE) sections of both IDC and DCIS were analyzed for the expression of various proteins using IHC kit from Biogenex, CA, USA as per the protocol. The chromogen used was DAB, counter stain used was hematoxylin and the tissue sections were mounted with DPX and observed under a compound microscope (Nikon, Tokyo, Japan).

### Generation of MAF or CAF

The CAFs isolated from IDC and DCIS patient tissues were co-cultured for 5 days in cm from HCC1937 (cmHCC1937) and HCC1937/wtBRCA1 (cmHCC1937/wtBRCA1) for the generation of MAF and CAF respectively and characterized.

### CCL5 and Ezrin inhibition

For CCL5 inhibition, met-RANTES (R&D systems, MN, USA), a recombinant protein, which is shown to be a partial agonist in the monocyte chemotactic assays, was used. The cells were treated with 100 ng metCCL5 and the inhibition of CCL5 was confirmed by qRT-PCR. Cancer cells or CAFs cultured in presence of cmCAFs or cmHCC1937 and cmHCC1937/wt BRCA1 respectively were treated with or without metCCL5 for 48 h and then used for further analysis.

For Ezrin inhibition, pBS/U6 ezrin siRNA, which was a gift from Philip Hinds (Addgene plasmid # 8945) were used for transiently transfecting cancer cells/CAFs using lipofectamine reagent. The inhibition was confirmed with qRT-PCR. Further ELISA, migration and invasion assays were done.

### ELISA

MAF and CAF were treated with CCL5 inhibitor, metCCL5 or transfected with EzrinsiRNA for 48 h. The cm collected from these cells were analyzed for CCL5 using the Human Rantes Elisa kit (Calbiochem, CA, USA) as per the kit protocol.

### Statistical analysis

All the experiments were repeated at least thrice and expressed as mean ± S.D from at least three independent experiments. Error bars were given on the basis of calculated S.D values. The two tailed students t-test and ANOVA was used to test the probability of significant differences between different experimental groups. Statistical significance was defined as *p ≤ 0.05 and **p ≤ 0.005 and as ***p ≤ 0.001.

## Electronic supplementary material

Supplementary Information
