# Supplementary material for: Brcal Defective Breast Cancer Cells Induce in vitro Transformation of Cancer Associated Fibroblasts (CAFs) to Metastasis Associated Fibroblasts (MAF)
Source: Sci Rep. 2018 Sep 17;8:13903. doi: 10.1038/s41598-018-32370-w (PMC6141525; doi:10.1038/s41598-018-32370-w)
Supplement: Supplementary file 1 — Supplementary Information [file 41598_2018_32370_MOESM1_ESM.pdf]

<u>BRCA1 DEFECTIVE BREAST CANCER CELLS INDUCE *IN-VITRO* TRANSFORMATION OF CANCER ASSOCIATED FIBROBLASTS (CAFs) TO METASTASIS ASSOCIATED FIBROBLASTS (MAF)</u>

Sreelatha K Hemalatha[1], Satheesh Kumar Sengodan[1¶], Revathy Nadhan[1], Jithin Dev[1], Reshma R Sushama[1], Veena Somasundaram[1¶¶], Ratheeshkumar Thankappan[1], Arathi Rajan[1], Neetha Rajan Latha[1], Geetu Rose Varghese[1], Arun Peter Mathew[2], Thara Somanathan[2¶], Priya Srinivas[1*]

[1]Cancer Research Program, Rajiv Gandhi Centre for Biotechnology, Thiruvananthapuram, Kerala, India, Current affiliation: [1¶]Mouse Cancer Genetics Program, Center for Cancer Research, National Cancer Institute, Frederick, MD 21702-1201, [1¶¶]Cancer and Inflammation Program, Center for Cancer Research, National Cancer Institute, Frederick, MD 21702-1201, [2]Department of Surgical Oncology, Regional Cancer Centre, Thiruvananthapuram, Kerala, India, [2¶]Department of Pathology, Regional Cancer Centre, Thiruvananthapuram, Kerala, India.

*Correspondence to: Dr. Priya Srinivas, Cancer Research Program, Rajiv Gandhi Centre for Biotechnology, Thycaud PO, Thiruvananthapuram 695 014, Kerala, India. Tel: 91-471-2529495; Fax: 91- 471- 23 48 096. E-mail: priyasrinivas@rgcb.res.in.

Supplementary files

Universal primer sequence for mycoplasma testing:

Forward primer: GTG GGG AGC AAA YAG GAT TAG A
Reverse primer: GGC ATG ATG ATT TGA CGT CRT

TCGA data analysis.

cBioportal for cancer genomics was used to analyze the expression of CCL5, Moesin, Radixin and BRCA1 in breast cancer tissues samples (http://www.cbioportal.org/) and the same represented as Supplementary figure S4.

Supplementary table S1:

Fold expression of ERM and CCL5 in 4 IDC and 3 DCIS samples by qRT-PCR analysis

	Fold Expression in MAF				Fold expression in CAF			
**Sample No**	**Ezrin**	**Radixin**	**Moesin**	**CCL5**	**Ezrin**	**Radixin**	**Moesin**	**CCL5**
**Tumor type: IDC**								
Sample 1	3.65451	2.56084	6.57778	24.06578	3.731907	0.8217	1.51115	1.56637
Sample 2	4.33211	6.14157	31.8752	953.5052	0.283476	18.6455	1.65983	624.087
Sample 3	2742.23	831.548	20.078	8849.195	0.421534	0.3316	0.35028	0.75441
Sample 4	13.2419	2.43815	34.9049	39.0012	0.803263	3.45725	0.47299	0.27035
**Average**	**690.86**	**210.67**	**23.359**	**2466.44**	**1.31004**	**5.814**	**0.9986**	**156.67**
**Tumor type: DCIS**								
Sample 6	0.66553	1.83004	0.78025	7.148032	12.91767	1.55038	1.15954	3.06696
Sample 7	0.25186	0.30871	0.34448	0.64627	0.442333	0.73947	0.40488	20.5466
Sample 8	0.58625	1.54531	0.10905	1.7266	0.451793	0.28507	0.30141	11.7264
**Average**	**0.5012**	**1.228**	**0.4113**	**3.17363**	**4.60393**	**0.8583**	**0.6219**	**11.78**

Supplementary table S2:

Antibodies used for the study

Antibody	Company	Catalogue no
αSMA	Thermo scientific	MA5-11547
FAP	Thermo scientific	PA5-27773
F-actin	Thermo scientific	MA-1 80729
CD10	Thermo scientific	MA5-14050
FSP	Cell Signaling Technology	#13018
Vimentin	Cell Signaling Technology	#5741
E-cadherin	Cell Signaling Technology	#3195
Ezrin	Cell Signaling Technology	#3145
Radixin	Cell Signaling Technology	#2636
Moesin	Cell Signaling Technology	#3150
CCL5	Cell Signaling Technology	#2987
Caveolin-1	Cell Signaling Technology	#3267
SDF-1	Cell Signaling Technology	#3740
MMP9	Cell Signaling Technology	#13667
γH2A.x	Cell Signaling Technology	#2577S
BRCA1	Cell Signaling Technology	#9010
N-cadherin	Santacruz	sc-31030
Cytokeratin 14	Santacruz	sc-53253
Cytokeratin 18	Santacruz	sc-32329
βactin	Santacruz	sc-47778
Bovine anti-goat IgG HRP	Santacruz	sc-2378
Mouse anti-rabbit IgG HRP	Santacruz	sc-2357
Goat anti-mouse IgG HRP	Santacruz	sc-2005
Donkey anti-mouse IgG PE	Santacruz	sc-3744
Mouse anti-goat IgG FITC	Santacruz	sc-2356
Goat anti-rabbit IgG FITC	Santacruz	sc-2010

Supplementary table S3:

Primer sequences used for the study

Gene	Direction	Sequences
α SMA	Forward (5’-3’)	CAGGGCTGTTTTCCCATCCAT
	Reverse (5’-3’)	ACGTAGCTGTCTTTTTGTCCC
FAP	Forward (5’-3’)	ACGGCTTATCACCTGATCGG
	Reverse (5’-3’)	AATTGGACGAGGAAGCTCATTT
FSP1	Forward(5’-3’)	GATGAGCAACTTGGACAGCAA
	Reverse (5’-3’)	CTGGGCTGCTTATCTGGGAAG
SDF-1	Forward (5’-3’)	TTTGAGAGCCATGTCGCCA
	Reverse (5’-3’)	TGTCTGTTGTTGCTTTTCAGCC
Caveolin-1	Forward (5’-3’)	ACCCACTCTTTGAAGCTGTTG
	Reverse (5’-3’)	GAACTTGAAATTGGCACCAGG
Vimentin	Forward (5’-3’)	GACAATGCGTCTCTGGCACGTCTT
	Reverse (5’-3’)	TCCTCCGCCTCCTGCAGGTTCTT
CCL5	Forward (5’-3’)	TACCATGAAGGTCTCCGC
	Reverse (5’-3’)	GACAAAGACGACTGCTGG
E-cadherin	Forward (5’-3’)	GAAGGTGACAGAGCCTCTGGAT
	Reverse (5’-3’)	GATCGGTTACCGTGATCAAAATC
N-cadherin	Forward (5’-3’)	ACAGTGGCCACCTACAAAGG
	Reverse (5’-3’)	CCGAGATGGGGTTGATAATG3
Fibronectin	Forward (5’-3’)	GAAGCTCTCTCTCAGACAACCA
	Reverse (5’-3’)	GCCCACGGTAACAACCTCTT
Ezrin	Forward (5’-3’)	GTTTTCCCCAGTTGTAATAGTGCC
	Reverse (5’-3’)	TCCGTAATTCAATCAGTCCTGC
Radixin	Forward (5’-3’)	CGAGGAAGAACGTGTAACCGAA
	Reverse (5’-3’)	TCTTGGTTTCATCTCTGGCTTG
Moesin	Forward (5’-3’)	TGTAAACCAGAGAGCTGCTGG
	Reverse (5’-3’)	GAAGAGCACACATGAGACAGAGAA

Supplementary figure legends

Supplementary figure S1: (A) Phase contrast images of CAFs and NFs (Magnification: 10X) (B) Immunofluorescence analysis for the expression of αSMA, FSP, CD10, Vimentin, Cytokeratin14 (K14) in CAFs and NFs (Magnification: 60X). (C) Flow cytometric analysis for the expression of CD10 and ESA in CAFs and NFs. Right panel is the graphical representation for the same. (D) Western blot analysis for αSMA, Vimentin, Cytokeratin 18 (K18) and FAP in CAFs, NFs, HCC1937 and MDAMB-231 cells. β- actin was the loading control. Quantitation for the western blot is given on the right panel. Error bars in the graphs represent standard deviation.

Supplementary figure S2: (A) qRT-PCR analysis for the expression of BRCA1, Caveolin-1 and p53 in breast cancer cells grown with cmCAFs. (B) Immunofluorescence analysis for the expression of Caveolin-1 in HCC1937 and HCC1937/wt BRCA1 in presence or absence of cmCAFs (Magnification: 60X). (C) Proliferation rate of CAFs assessed by conditioned media from MCF-7, MDA-MB-231 and MX1 cells by cell proliferation assay (D) Upper panel shows the phase contrast images representing the morphological appearance of CAFs co-treated with cmHCC1937 and cmHCC1937/wt BRCA1 and the lower panel represents magnified images (60X) of similar treated CAFs. Error bars in the graphs represent standard deviation.

Supplementary figure S3: (A) Flow cytometric analysis for the expression of CD10 and ESA in CAFs co-cultured with cmHCC1937 and cmHCC1937/wt BRCA1 for 5 days along with the graph representing its quantitation. (B) qRT-PCR analysis for the expression of αSMA, FAP, FSP, Vimentin, SDF-1, BRCA1, Caveolin-1, and p53 in CAFs co-cultured with cmHCC1937 and cmHCC1937/wt BRCA1. (C) Immunohistochemical analysis for the expression of Ezrin, Radixin, Moesin and CCL5 in DCIS, normal and LIF tissues (Magnification: 40X). Black arrows indicate the expression of proteins in CAFs (n=3). Error bars in the graphs represent standard deviation.

Supplementary figure S4: (A) Table representing the TCGA data analysis of breast cancer tissues using the cBioPortal for Cancer Genomics and the right panel shows the representative heatmap image from an 817 breast cancer tissue sample data set. (B) qRT-PCR analysis for the fold expression of CCL5 in HCC1937 and HCC1937/wt BRCA1 treated with metCCL5. (C) Proliferation of HCC1937 and HCC1937/wt BRCA1 in the presence of mCCL5 (metCCL5),

mCCL5 and cmCAFs. (D) Migration analysis and its quantitation of breast cancer cells grown in cmCAFs treated with metCCL5 (E) qRT-PCR analysis for the fold expression of Ezrin, Radixin and Moesin in HCC1937 and HCC1937/wt BRCA1 cells transfected with Ezrin siRNA plasmid. Error bars in the graphs represent standard deviation.

Supplementary figure S5: Schematic diagram representing the generation of MAF from CAFs by BRCA1 deficient cancer cells. Cancer cells, once they are initiated from the normal epithelial cells are capable of converting NFs to CAFs by the secretion of various growth factors. CAFs thus formed in turn influences the cancer cells for increased progression. Further these cancer cells due to genomic instability accumulate mutations, at one point genes that control the motility (eg., BRCA1 which has interaction with the cytoskeletal protein ERM) get deregulated initiating metastasis. This will result in the generation of MAF that assist cancer cells for metastasis. When BRCA1 is mutated it can generate a distinct population of CAFs, MAF which are more mesenchymal in nature and overexpress the ERM proteins that will result in the increase in cell motility.

Supplementary figure S6: Original blots for western blot analysis in the main Figure 1H and Figure 2B.

Additional information on methodology

Isolation and Culturing of CAFs

CAFs used in the study were isolated from human breast cancer tissue samples and NFs were isolated from the non-malignant part of the same breast tissue which was about 5-8 cm apart from the cancer tissue by collagenase digestion method. The collected tissue mass was washed with 1X PBS to remove blood and other debris and then cut into small pieces using surgical blades. The cut tissues were digested with 1X collagenase in 1X DMEM and incubated at 37°C for 16-20 h. After incubation the digested tissues with medium was centrifuged at 100 g for 1 minute to separate the supernatant. The supernatant was again centrifuged at 400 g for 5 minutes to separate the CAFs as pellets, which was then seeded into culture flask with 20% DMEM containing 2X antibiotics and incubated at 37°C supplemented with 5% CO_2. Explant cultures with the undigested tissues were also performed, so as to obtain maximum CAFs for the experiments from the available tissues. CAFs which have grown out from the explants cultures

were isolated by differential trypsinization method and grown in 10% DMEM with FBS at 37^oC supplemented with 5% CO_2.

Characterization of isolated CAFs/NFs:

<u>Immunofluorescence analysis</u>: Cells were seeded in coverslips in 24 well plates (about 50% confluent) were grown for 24 hours at 37^oC in a CO_2 incubator. Afterwards the cells were fixed with 4 % paraformaldehyde for 20 minutes in ice and then permeabilized with 0.2 % Triton-X-100 for 10 minutes. The cells were then blocked using 1 % BSA in PBST for 30 minutes. Further primary antibodies diluted in 1 % BSA in PBST was added and incubated at 4^oC overnight. After removing primary antibody, cells were washed with 1X PBST, incubated with specific FITC/PE conjugated secondary antibodies and incubated for 1 hour at room temperature. Again the cells were washed with 1X PBST and incubated with the nucleic acid stain, DAPI for 10 minutes. The coverslips with the cells were then mounted to glass slides with glycerol PBS, sealed with DPX and observed under the confocal microscope.

Co-culture studies:

In the indirect co-culture method, breast cancer cell lines, HCC1937, HCC1937/wt BRCA1, MCF-7 and MDAMB-231 were allowed to grow in the presence of cmCAFs or CAFs were allowed to grow in the conditioned medium from breast cancer cells for 3 and 5 days respectively in 100 mm dishes. For collecting conditioned media, cells grown in their respective medium were washed with 1X PBS at 70% confluency and then incubated with SFM. After 24 hours the supernatant was collected, centrifuged to remove debris, filtered using 2 µm pore size syringe filter and freeze stored until use. For indirect co-culture, either of the cells were seeded onto 100 mm dishes and incubated at 37^oC for 24 hours with their respective medium for proper attachment and growth. Afterwards, the cells were incubated with the conditioned medium as indicated in each experiment. After incubation the cells were used for different assays.

For direct co-culture, both the CAFs and the breast cancer cells in the ratio 1:2 were allowed to grow together under the same condition as indicated of indirect co-culture method for 5 days. Briefly, at first the CAFs were seeded on to the 100 mm culture dishes, allowed to attach by incubating it at 37^oC in a CO_2 incubator. Once these cells attaches, the breast cancer cells were seeded and incubated under the same condition as above.

Supplementary Figure S1

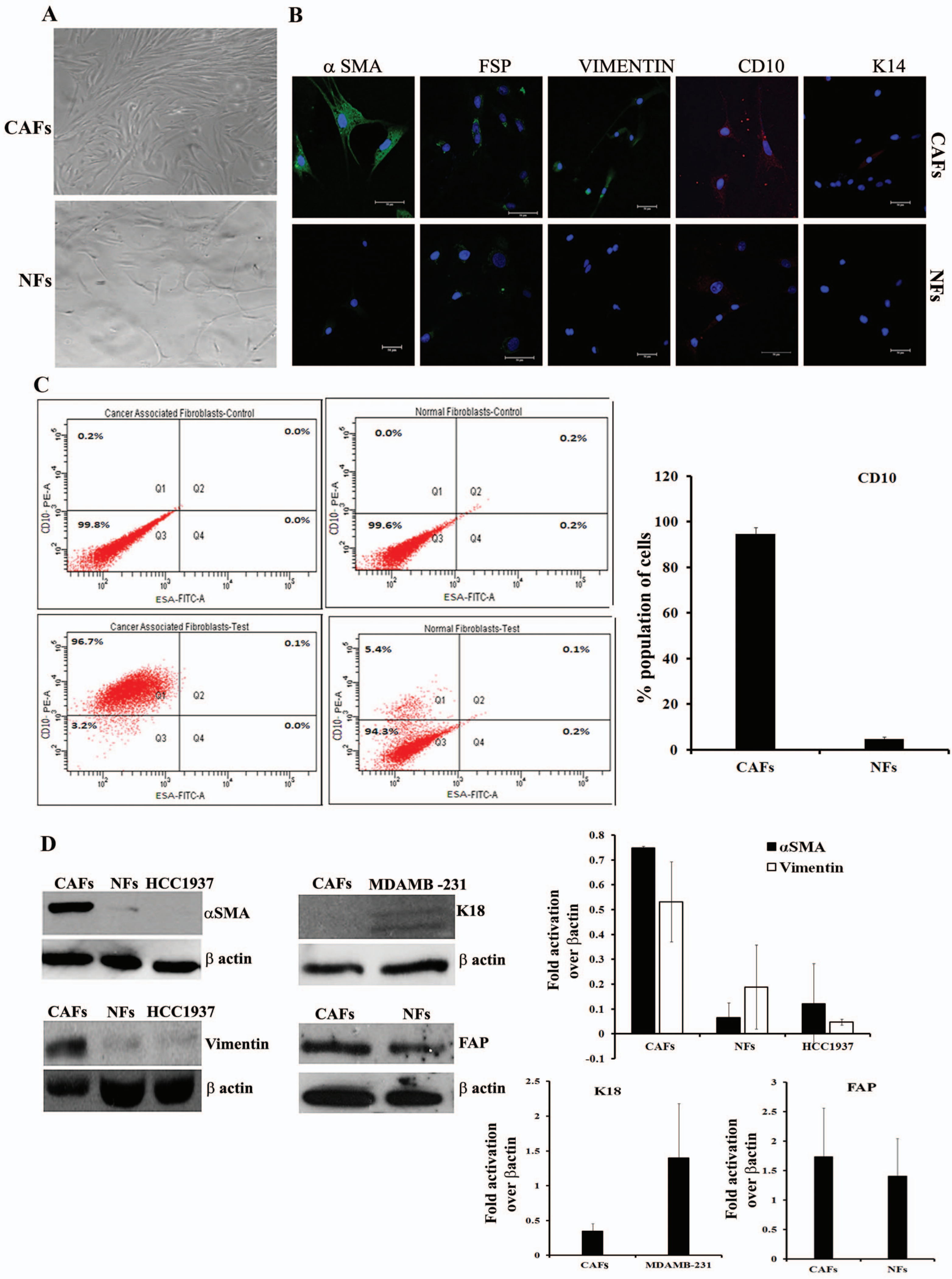

Supplementary Figure S2

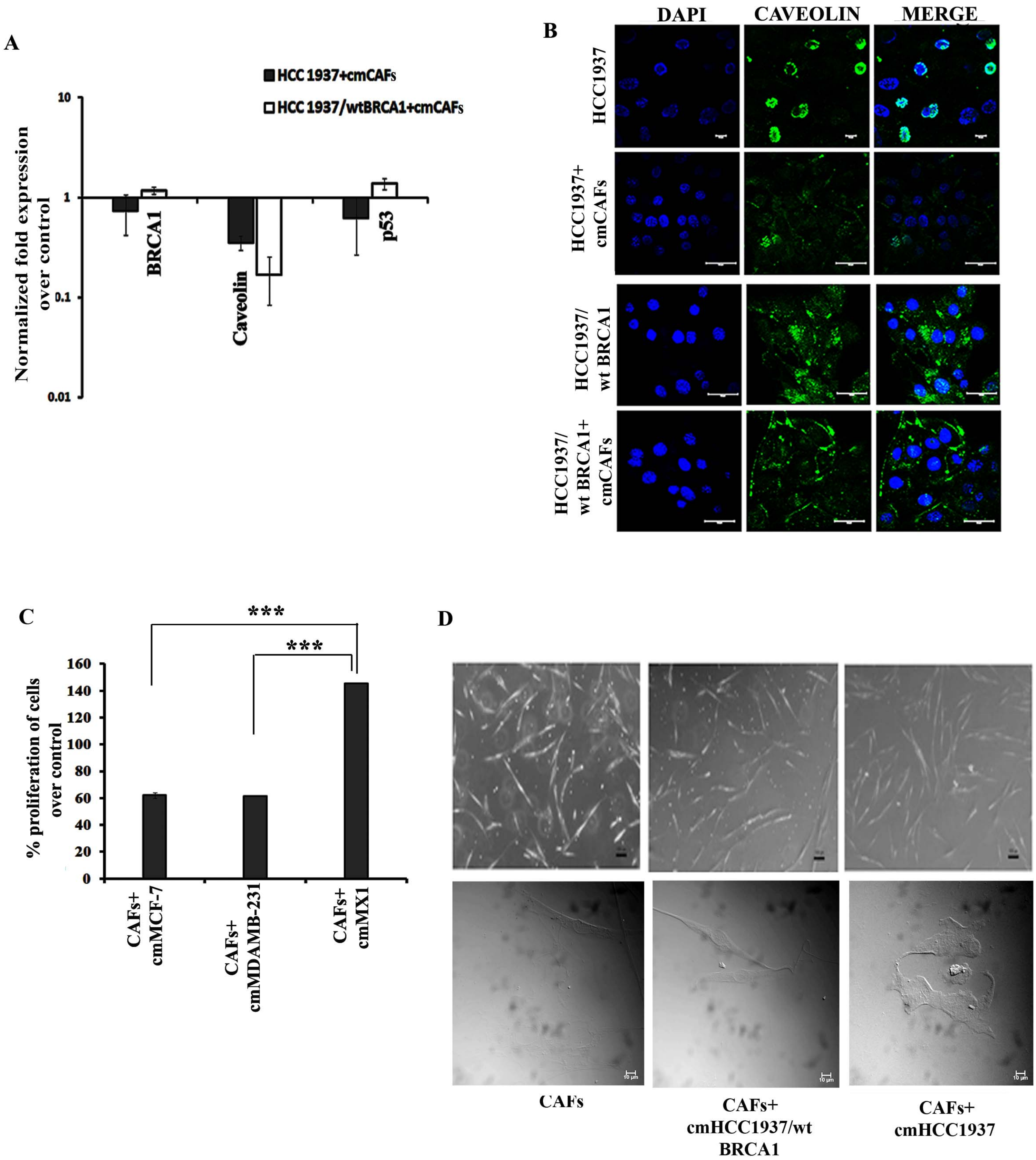

Supplementary Figure S3

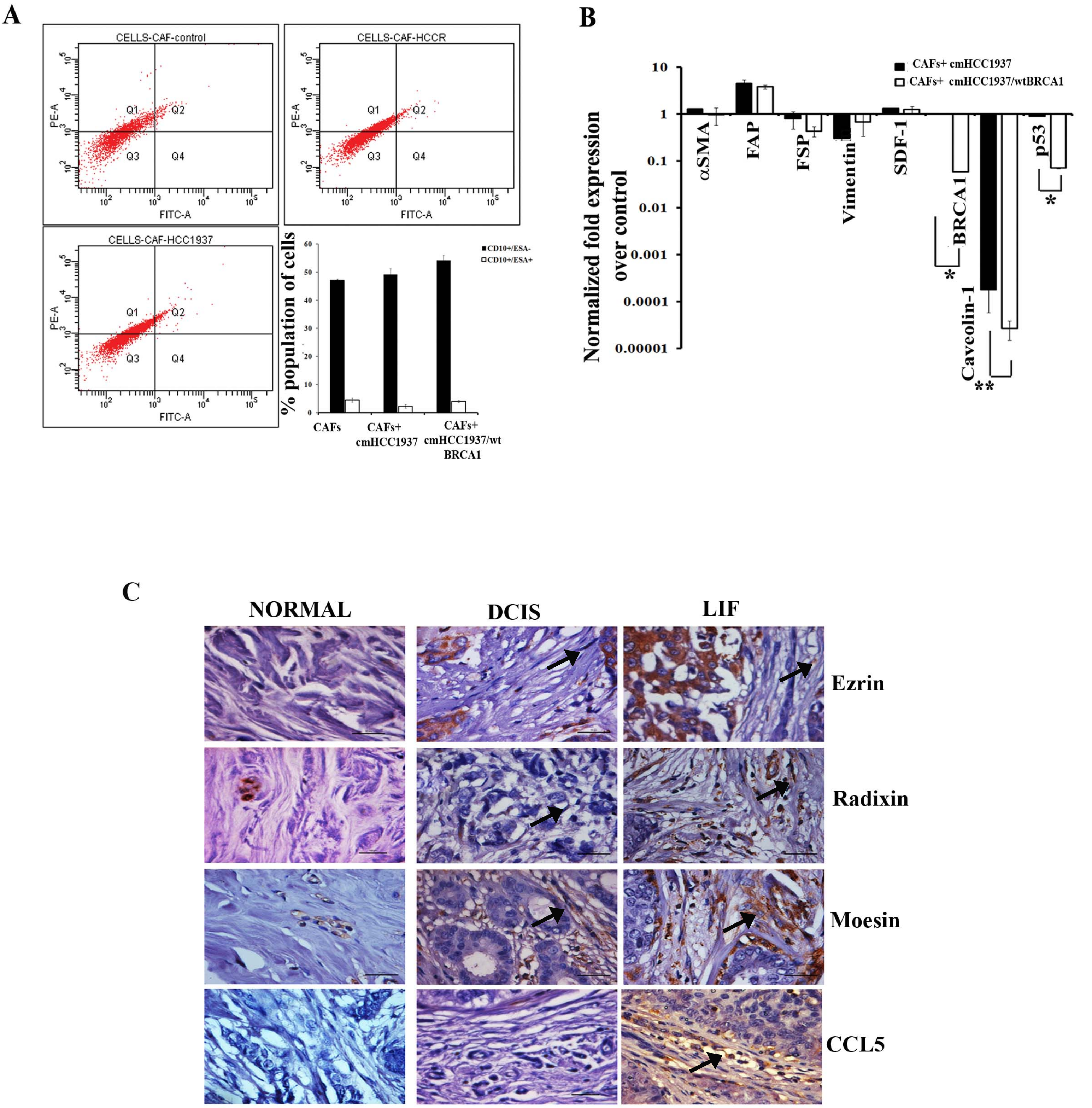

Supplementary Figure S4

A

Sl. No	Data set	BRCA1 Vs CCL5		BRCA1 Vs MSN		BRCA1 Vs RDX	
		Pearson	Spearman	Pearson	Spearman	Pearson	Spearman
1	Breast cancer (METABRIC, Nature 2012 & Nature communications 2016, n= 2051)	-0.155	-0.163	-0.190	-0.192	-0.162	-0.160
2	Breast Invasive Carcinoma (TCGA, Provisional, n= 1105)	-0.107	-0.202	-0.110	-0.147	0.119	0.111
3	Breast invasive Carcinoma (TCGA, Nature 2012,n= 825)	-0.135	-0.134	-	-	-0.101	-0.110
4	Breast Invasive Carcinoma (TCGA, Cell 2015, n= 817)	-0.111	-0.205	-0.142	-0.183	0.093	0.079

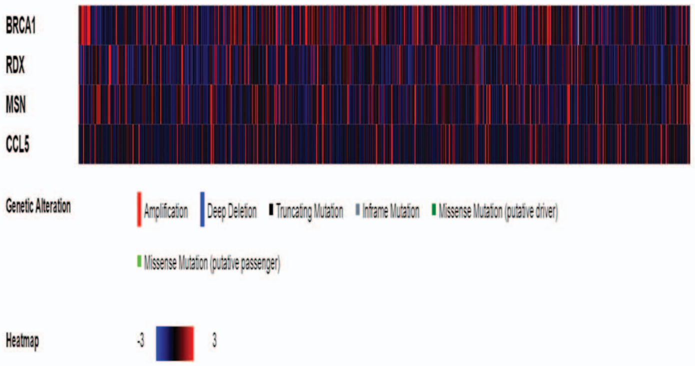

B

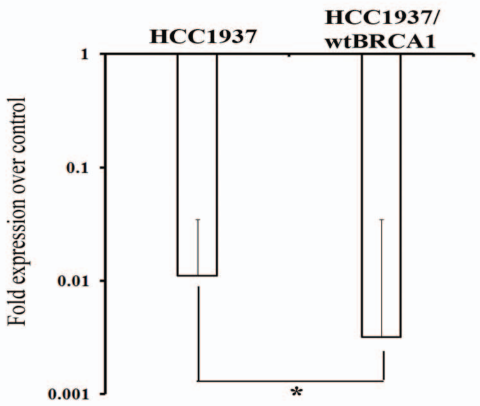

C

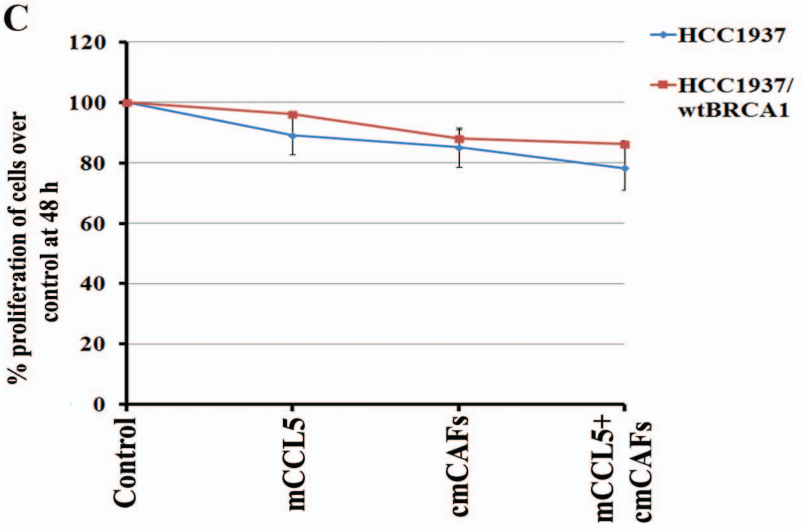

D

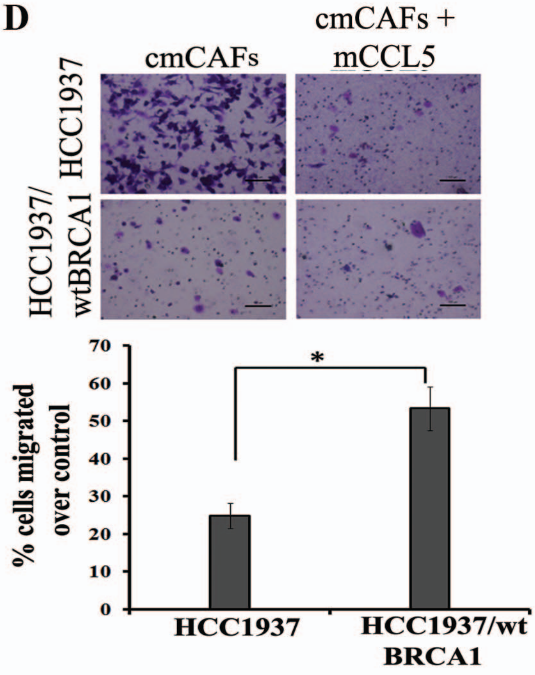

E

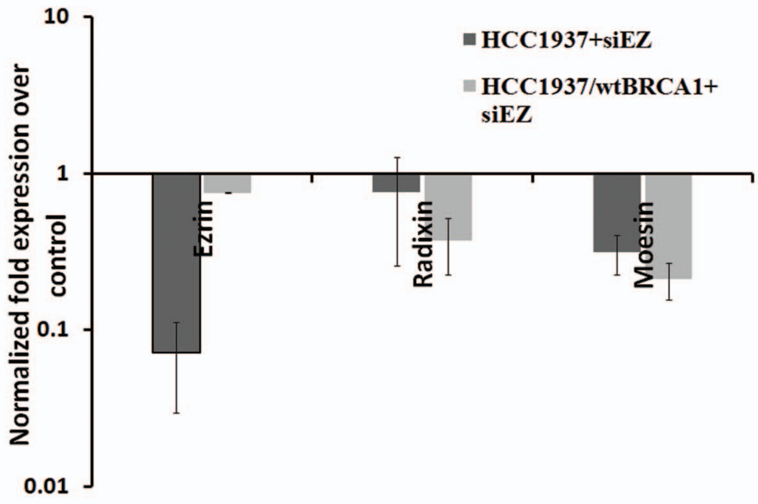

Supplementary Figure S5

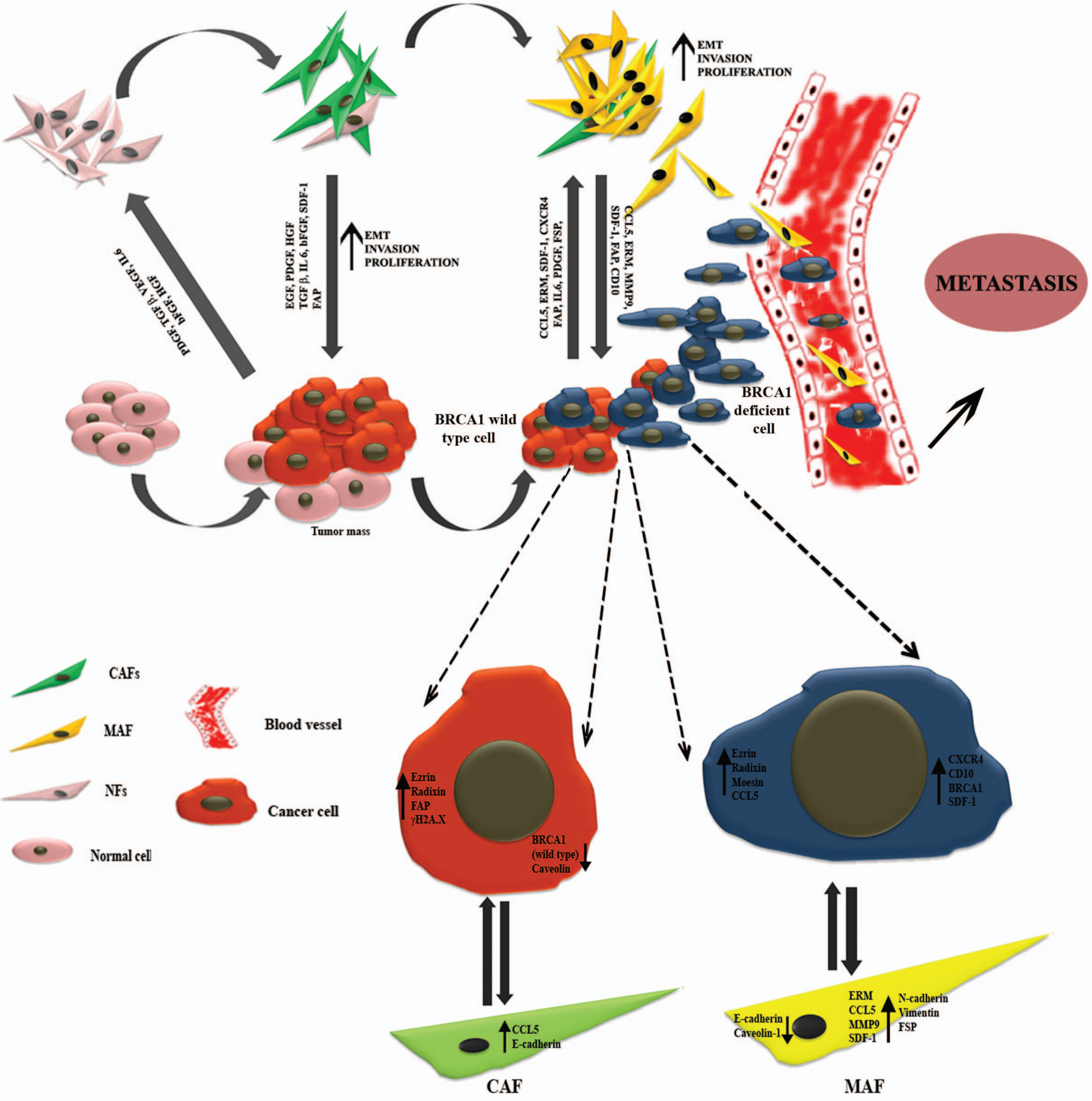

Supplementary Figure S6

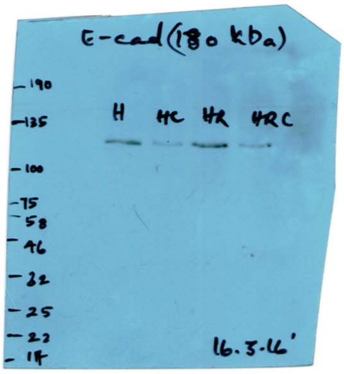

E-cadherin

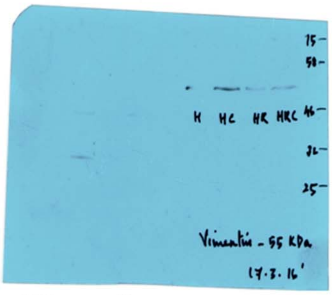

Vimentin

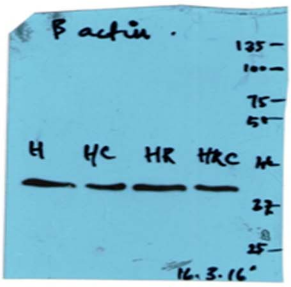

β actin for E-cadherin and Vimentin

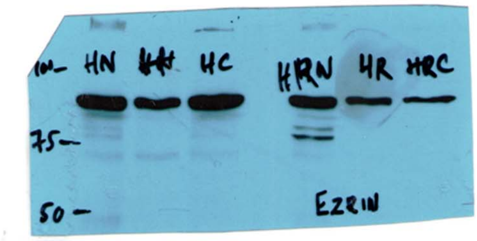

Ezrin

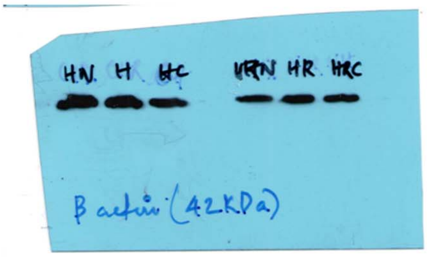

β actin for Ezrin

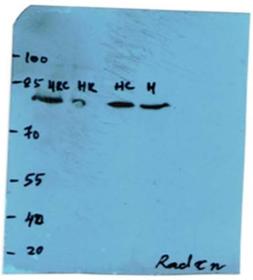

Radixin

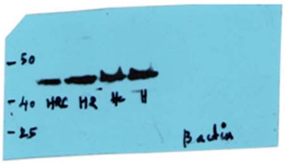

β actin for Radixin

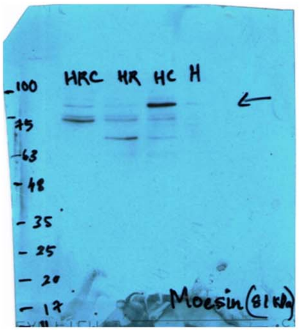

Moesin

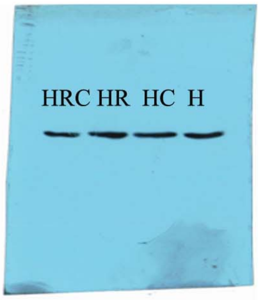

β actin for Moesin
